# Antimicrobial Peptides With Antibiofilm Activity Against *Xylella fastidiosa*

**DOI:** 10.3389/fmicb.2021.753874

**Published:** 2021-11-08

**Authors:** Luís Moll, Esther Badosa, Marta Planas, Lidia Feliu, Emilio Montesinos, Anna Bonaterra

**Affiliations:** ^1^Laboratory of Plant Pathology, Institute of Food and Agricultural Technology-CIDSAV-XaRTA, University of Girona, Girona, Spain; ^2^LIPPSO, Department of Chemistry, University of Girona, Girona, Spain

**Keywords:** *Xylella fastidiosa*, bactericidal peptides, antibiofilm peptides, biofilm production, planktonic cells

## Abstract

*Xylella fastidiosa* is a plant pathogen that was recently introduced in Europe and is causing havoc to its agriculture. This Gram-negative bacterium invades the host xylem, multiplies, and forms biofilm occluding the vessels and killing its host. In spite of the great research effort, there is no method that effectively prevents or cures hosts from infections. The main control strategies up to now are eradication, vector control, and pathogen-free plant material. Antimicrobial peptides have arisen as promising candidates to combat this bacterium due to their broad spectrum of activity and low environmental impact. In this work, peptides previously reported in the literature and newly designed analogs were studied for its bactericidal and antibiofilm activity against *X. fastidiosa*. Also, their hemolytic activity and effect on tobacco leaves when infiltrated were determined. To assess the activity of peptides, the strain IVIA 5387.2 with moderate growth, able to produce biofilm and susceptible to antimicrobial peptides, was selected among six representative strains found in the Mediterranean area (DD1, CFBP 8173, Temecula, IVIA 5387.2, IVIA 5770, and IVIA 5901.2). Two interesting groups of peptides were identified with bactericidal and/or antibiofilm activity and low-moderate toxicity. The peptides **1036** and **RIJK2** with dual (bactericidal–antibiofilm) activity against the pathogen and moderate toxicity stand out as the best candidates to control *X. fastidiosa* diseases. Nevertheless, peptides with only antibiofilm activity and low toxicity are also promising agents as they could prevent the occlusion of xylem vessels caused by the pathogen. The present work contributes to provide novel compounds with antimicrobial and antibiofilm activity that could lead to the development of new treatments against diseases caused by *X. fastidiosa.*

## Introduction

*Xylella fastidiosa* is a Gram-negative xylem-inhabiting bacterium that causes important plant diseases that pose great threats to the agriculture worldwide (Purcell, 2013). This pathogen was first detected in California in grapevines causing Pierce’s disease (Alston et al., 2013). It is also responsible for other plant diseases such as citrus variegated chlorosis (Rapicavoli et al., 2018) and almond leaf scorch disease. In 2013, it was introduced in Italy and is spreading through the Mediterranean region causing a new disease named olive quick decline syndrome (EFSA, 2013). The increasing dissemination of *X. fastidiosa* can be related to many factors, such as climate conditions optimal for its growth, its easy spread through insect vectors from the *Cicadellidae* (sharpshooter leafhoppers) or the *Aphrophodridae* (meadow spittlebug) families, and the huge number of hosts that it can infect (Almeida and Nunney, 2015; EFSA, 2015; Strona et al., 2017). Therefore, this pathogen could cause havoc in the agricultural economy of countries that are important global producers of olives, citrus, almonds, and grapes, such as Italy, Spain, France, and Greece (Food and Agriculture Organization of the United Nations, 2019).

Since *X. fastidiosa* inhabits xylem vessels in host plants, biofilm formation is the main pathogenic mechanism for the symptomatology of plants infected by this pathogen (Cardinale et al., 2018). Once *X. fastidiosa* is inoculated into the host xylem vessels by an insect vector, the cells first remain in a planktonic stage and then are reversibly attached to the vessels’ surface. Next, cells are irreversibly embedded in a self-produced matrix of extracellular polymeric substances (EPS) leading to the formation of the biofilm (Cattò et al., 2019). Eventually, the architecture of this biofilm matures and reaches its maximum complexity occluding the xylem vessels, blocking the sap flow and depriving the plants of water and nutrition (Martelli et al., 2016). Finally, cells detach from the biofilm and become planktonic again, being able to disperse to other areas of the plant (Mendes et al., 2016). In this planktonic state, cells can be acquired by vectors when they feed upon the xylem of infected plants spreading the pathogen to healthy plants.

At present, most of the measures adopted to manage the diseases caused by *X. fastidiosa* are aimed to limit the spread of the bacterium. Some of these strategies are related to agricultural practices such as the application of insecticides to control the vector population and the eradication of infected plants (EFSA, 2016). Europe is migrating to a more sustainable agriculture model so many chemical compounds used in the past to control bacterial plant pathogens have been prohibited or restricted to be used on field (Navarrete and De La Fuente, 2014; ECDC, EFSA and EMA, 2015; EFSA, 2016). Nevertheless, different approaches have been studied consisting of new chemicals and biological control strategies. Some chemical compounds such as *N*-acetyl-L-cysteine (NAC) in citrus plants (Muranaka et al., 2013), copper (II) sulfate in tobacco plants (Ge et al., 2020), and menadione, benzethonium chloride, and abscisic acid in grapevines (Meyer and Kirkpatrick, 2011; Zhang et al., 2019) seem to be effective in greenhouse conditions. Moreover, the antibiotic oxytetracycline along with three other compounds, like NAC, a bioactive detergent composed of plant oil extracts, and a Zn, Cu, and citric acid fertilizer, showed potential to be used to control *X. fastidiosa* diseases in almond (Amanifar et al., 2016) and olive orchards (Dongiovanni et al., 2017; Scortichini et al., 2018; Bruno et al., 2021), respectively. Other strategies that have been studied involve the use of the endophyte *Paraburkholderia phytofirmans* (Baccari et al., 2019), avirulent *X. fastidiosa* strains (Hao et al., 2017), and lytic phages (Das et al., 2015) as biological control agents. Although the results obtained in most of these trials were positive, no strategy was able to completely cure plants infected by *X. fastidiosa*. Therefore, there is still a need to find efficient compounds and eco-friendly alternatives that comply with the European environmental regulations.

Antimicrobial peptides are a class of peptides that could be considered as promising candidates to control *X. fastidiosa.* In general, they exhibit high antibacterial activity and low toxicity (Guell et al., 2011; Li et al., 2020; Liang et al., 2020). In addition, they are not persistent compounds and resistance to them in pathogens is difficult to emerge since their mechanism of action mainly involves cell membrane disruption (Yeaman, 2003; Brogden, 2005; Peschel and Sahl, 2006; Von Borowski et al., 2018). Up to now, few antimicrobial peptides with activity against *X. fastidiosa* have been reported. In particular, indolicidin and magainin 2 have shown activity against several strains with minimum inhibitory concentration (MIC) between 8 and 64 μM (Li and Gray, 2003; Kuzina et al., 2006; Fogaça et al., 2010). Moreover, we recently identified the bactericidal peptides **BP171** and **BP178**, which are active against several *X. fastidiosa* strains with a reduction in viability approximately 3.6 log at 12.5 μM (Baró et al., 2020a, b).

It is worth mentioning that, despite the fact that biofilm is the main virulence factor for X. fastidiosa, there have not been reported peptides able to inhibit its formation. At the moment, only a few non-peptidic compounds have been reported to present some antibiofilm activity against this pathogen such as the previously mentioned, NAC and the Zn, Cu, and citric acid fertilizer, DOX-derived oxylipins, and phenolic compounds such as gallic acid and epicatechin (Muranaka et al., 2013; Lee et al., 2020; Scala et al., 2020; Tatulli et al., 2021). Nevertheless, peptides with antibiofilm activity against other Gram-negative bacteria (Escherichia coli, Pseudomonas aeruginosa, Acinetobacter baumannii, Klebsiella pneumoniae, and some species of Salmonella) or sequences with both antibacterial and antibiofilm activity have been widely described. These peptides could be considered good candidates to be tested against X. fastidiosa. Among these potential candidates, peptides from the family of **RR** showed antibacterial and antibiofilm activity against multidrug resistant clinical strains (Mohamed et al., 2017). Other peptides that displayed both antibacterial and antibiofilm activity are the LL-37 derivative **KR-12-a5** and the peptide **SB056** (Batoni et al., 2016; Kim et al., 2017). De La Fuente-Núñez et al. described antimicrobial peptides that target biofilm formation, including **LJK2** and its retro-inverso analog **RIJK2**, and the innate defense regulator **IDR-1018** (De La Fuente-Núñez et al., 2015). These authors also identified the small cationic antimicrobial peptide **HH15** and its analogs **1026**, **1029**, **1036,** and **1037**, which displayed antibacterial and/or antibiofilm activities (De La Fuente-Núñez et al., 2012). All these peptides share the consensus sequence FRIRVRV-NH_2_ (**FV7**), which was later proven to be active and used to design the conjugate **R-FV7-I16** (Xu et al., 2014). Scorpion venom peptides **AamAP1** and **HP1404** have also been described to display interesting biological properties and their sequence has served as basis for the design of new analogs, including **AamAP-S1**, **HP1404-T1D**, and **HP1404-T1E** (Almaaytah et al., 2012; Kim et al., 2018).

Based on these considerations, the aim of the present work was to identify peptides able to control X. fastidiosa. First, the differential susceptibility of X. fastidiosa strains to antimicrobial peptides was assessed in order to select a representative strain to evaluate the activity of the peptides. Then, we synthesized the peptides mentioned above together with several new analogs, and tested them for their bactericidal and antibiofilm activity against X. fastidiosa. In addition, their effect on leaf infiltration in a tobacco plant model and their hemolytic activity were studied.

## Materials and Methods

### Synthesis of Peptides

Peptides (Table 1) were synthesized manually on solid phase using standard 9-fluorenylmethoxycarbonyl (Fmoc)/tert-butyl (tBu) strategy. A Fmoc-Rink-ChemMatrix resin (0.69 mmol/g), a PAC-MBHA resin (0.24 mmol/g), or a Fmoc-Rink-MBHA resin (0.56 mmol/g) was used as solid support. The Fmoc-Rink-ChemMatrix resin was selected for the synthesis of peptides containing more than 14 residues. The PAC-MBHA resin was employed to prepare C-terminal carboxylic acid peptides whereas the Fmoc-Rink-ChemMatrix and the Fmoc-Rink-MBHA resins served for C-terminal peptide amides. Peptide elongation was carried out through sequential steps of Fmoc removal and coupling of the corresponding amino acid as previously described (Caravaca-Fuentes et al., 2021; Oliveras et al., 2021). Once the peptide sequence was completed, each resulting peptidyl resin was treated with trifluoroacetic acid (TFA)/H_2_O/triisopropylsilane (TIS) (95:2.5:2.5). Peptidyl resins that contained tryptophan and/or arginines were treated with TFA/H_2_O/TIS/thioanisole/1,2-ethandithiol/phenol (81.5:5:1:5:2.5:5). Following TFA evaporation and diethyl ether extraction, the crude peptides were purified by reverse-phase column chromatography, lyophilized, analyzed by HPLC, and characterized by mass spectrometry.

**TABLE 1:** Sequences of the peptides and their previously described activities.

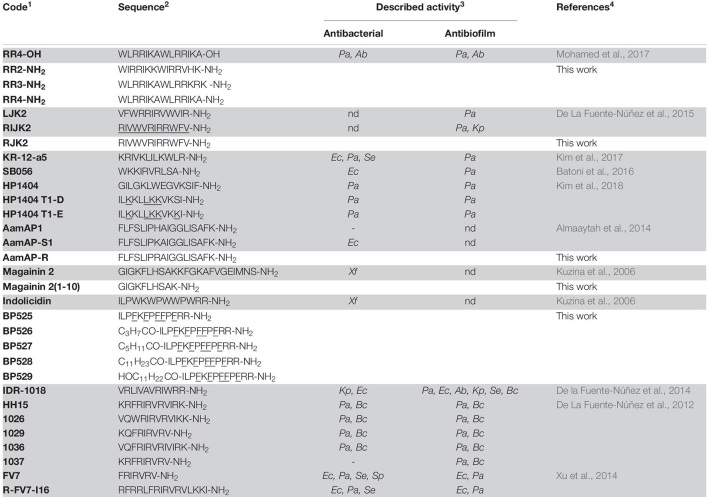

*^1^Peptides highlighted in gray have been previously described.*

*^2^Underlined amino acids stand for the corresponding D-isomer.*

*^3^Only activities described against Gram-negative bacteria are taken into consideration. Xf, Xylella fastidiosa; Ec, Escherichia coli; Pa, Pseudomonas aeruginosa; Ab, Acinetobacter baumannii; Kp, Klebsiella pneumoniae; Bc, Burkholderia cenocepacia; Se, Salmonella enterica subsp. enterica; Sp, Salmonella pullorum; nd, not determined; –, low or no activity.*

*^4^Each reference corresponds to the peptides highlighted in gray.*

### Bacterial Strains, Growth Conditions, and Characterization

All the experiments were carried out in officially authorized laboratories under biosafety level II+ under containment conditions according to European and Mediterranean Plant Protection Organization (EPPO) (EPPO, 2006) and the EU (EFSA PLH Panel, 2018). The X. fastidiosa strains used in this work were X. fastidiosa subsp. fastidiosa (Xff) Temecula (ATCC 700964), X. fastidiosa subsp. fastidiosa (Xff) IVIA 5387.2, X. fastidiosa subsp. fastidiosa (Xff) IVIA 5770, X. fastidiosa subsp. pauca (Xfp) DD1, X. fastidiosa subsp. multiplex (Xfm) CFBP 8173, and X. fastidiosa subsp. multiplex (Xfm) IVIA 5901.2 (Table 2). All strains were stored in Pierce disease broth (PD2, Davis, 1980) supplemented with glycerol (30%) and maintained at –80°C. When needed, strains were cultured in buffered charcoal yeast extract (BCYE) agar plates (Wells et al., 1981) at 28°C for 7 days. Afterward, colonies were scrapped and cultured in new BCYE media at 28°C for 7 additional days before being used in any of the experiments. When liquid cultures were required, PD3 broth (Davis et al., 1981) was used. Cell suspensions were prepared in sterile succinate-citrate-phosphate buffer (SCP) for bactericidal experiments or in sterile phosphate-buffered saline buffer (PBS) for biofilm experiments. The suspensions were adjusted to an optical density at 600 nm (OD_600_) of 0.32, which corresponds approximately to 10^8^ CFU/ml, which was confirmed by plate counting in PD2 modified with Gelrite^TM^ (9 g/l).

**TABLE 2:** Strains of Xylella fastidiosa used in this work.

Xylella fastidiosa	ST^1^	Strain/Origin^2^	Host and geographical origin	References
subsp. fastidiosa	1	Temecula (ATCC 700964)	Grapevine, California (United States)	Rodrigues et al., 2003
	1	IVIA 5387.2	Almond, Mallorca (Spain)	Baró et al., 2020b
	1	IVIA 5770	Grapevine, Mallorca (Spain)	Arias-Giraldo et al., 2020
subsp. pauca	53	DD1 (CFBP 8402)	Olive, Apulia (Italy)	Saponari et al., 2017
subsp. multiplex	41	CFBP 8173	Prunus, Georgia (United States)	Schaad et al., 2004
	6	IVIA 5901.2	Almond, Alicante (Spain)	Giampetruzzi et al., 2019

*^1^ST, sequence type.*

*^2^ATCC, American Type Culture Collection; IVIA, Instituto Valenciano de Investigaciones Agrarias; CFBP, Collection Française de Bactéries Associées aux Plantes.*

Growth curves of selected X. fastidiosa strains were performed by culturing a cell suspension prepared in 180 μl of PD3 medium (adjusted at an OD_600_ of 0.1) and mixed with 20 μl of water in 96-well plates (Nuclon^TM^ Delta Surface, Thermo Fisher Scientific, Spain). Three replicates of 10 wells were prepared for each strain. The microplates were incubated at 28°C under shaking (120 rpm) for 7 days and measures of OD_600_ were performed each day using the EPOCH2 TC microplate reader (BioTek, Winooski, United States). Background values of OD were subtracted from data and area under the growth curve (AUGC), specific growth rate, and doubling time were calculated for each replicate and strain.

Biofilm formation was quantified at the end of the growth curve experiment described above using the crystal violet dye according to the methods previously described (Zaini et al., 2009). The total growth, planktonic growth (cells in suspension), and biofilm formed (cells adhered to the well surface) were estimated by measuring OD. Planktonic cells were recovered from the media and transferred into new microplates and OD_600_ was measured. To quantify the biofilm formed, the original 96-well plate was rinsed gently with sterile distilled water three times, stained with 250 μl of crystal violet (0.1%) for 20 min, and rinsed with sterile distilled water three times to discard excess dye. Finally, crystal violet adhered to the biofilm was solubilized with 250 μl of a mixture of ethanol/acetone (4:6) for 10 min and a measure of OD_595_ was made. Two independent experiments of the biofilm formation were carried out with three replicates of 10 wells for each strain.

Time course of biofilm formation was assessed in order to select the best time for biofilm formation of Xff IVIA 5387.2. Different times of incubation (from 1 to 7 days) were tested. In each experiment, growth, planktonic cells, and biofilm formation were measured after the selected incubation period as described above.

### Bactericidal Activity

Bactericidal activity of the peptides was assessed by a test contact coupled with viable-quantitative PCR (v-qPCR) as previously described (Baró et al., 2020a). Sensitivity and amplification efficiency of the v-qPCR were evaluated for all studied strains. Briefly, standard curves were prepared using viable, dead (by heating them at 95°C for 20 min), or a mixture of viable and dead cells. Dilutions of a homogeneous cell suspension in SCP buffer (from 10^8^ to 5 × 10^2^ CFU/ml) of viable or dead cells to a total volume of 200 μl in DNA low binding tubes were prepared. Mixtures of viable cells with a constant number of dead cells (1 × 10^6^ CFU/ml) were also included to assess the influence of dead cells. Two sets of dilutions for viable, dead, or mixture were prepared and one of them was treated with PMAxx (VWR, Barcelona, Spain). Briefly, PMAxx was added at a final concentration of 7.5 μM, and samples were incubated for 8 min in the dark at room temperature following a photoactivation of 15 min (PMA-Lite^TM^ LED Photolysis Device, Biotium, CA, United States). DNA extractions of all samples were performed using the GeneJET Genomic DNA Purification Kit (Thermo Fisher Scientific, United States) following the specific protocol for Gram-negative bacterial suspensions and were analyzed in duplicate by a TaqMan-based qPCR assay based on the 16S rRNA sequence (Baró et al., 2020a). Then, a calibration curve for each strain with and without PMAxx was calculated by using cell concentration and C_T_ values, determined by qPCR. Three independent experiments were performed for each curve.

X. fastidiosa strains’ susceptibility to the peptide **BP171** was tested by a contact exposure test combined with v-qPCR against the six X. fastidiosa strains (Table 2) as previously described (Baró et al., 2020a). Briefly, the peptide was solubilized in sterile Milli-Q water to a stock concentration of 1 mM and filter sterilized through a 0.22 μM pore size filter. **BP171** was tested at a final concentration of 3.1 and 12.5 μM. Twenty microliters of the corresponding peptide dilution were mixed with 180 μl of a X. fastidiosa suspension, as described above. Three biological replicates for each concentration were performed and a non-treated control with sterile water instead of the peptide was included. Contact tests were incubated at room temperature for 3 h. Afterward, each tube was treated with PMAxx and was handled as previously described. The reduction in viability, expressed as log_10_ CFU/ml, was obtained by interpolating the C_T_ values from each sample against the respective standard curve for each strain and subtracting it from the non-treated control (Log_10_ (N_0_/N)).

The bactericidal activity of the selected peptides (Table 1) at 50 μM against Xff IVIA 5387.2 was determined as described above. Cecropin B (C1796, Merck, Spain) was also tested as reference control (Li and Gray, 2003). Highly active peptides (reduction in viability > 3 logs) were further tested at 12.5 and 3.1 μM to better characterize their bactericidal activity.

### Antibiofilm Activity

The effect of N-acetyl-L-cysteine (NAC; A9165, Merck, Spain) on biofilm formation of the studied strains was determined since it was previously described to reduce biofilm formation of X. fastidiosa (Muranaka et al., 2013). NAC was tested at a final concentration of 50 μM. Twenty microliters of NAC were mixed with 180 μl of a X. fastidiosa suspension in PD3 in 96-well plates, as previously described in this study. Three replicates of 10 wells were made for each strain. Microplates were incubated at 28°C for 5 days under continuous shaking (120 rpm). Finally, growth, planktonic cells and biofilm formation were measured as previously described. The ratio of biofilm formation was calculated according to the formula Oi/Oc, where Oi is the OD_595_ of the treatment and Oc is the OD_595_ of the non-treated control. The ratio of planktonic cells was calculated as described above but OD was measured at 600 nm.

To assess the antibiofilm activity of all the synthetized peptides, they were prepared as described in the bactericidal activity experiments. They were tested for antibiofilm activity at a final concentration of 50 μM against Xff IVIA 5387.2 as previously described in this study. Magainin 2 was tested at 12.5 μM and **RIJK2** and **1036** were tested at 3.1 μM to prevent the influence of their antimicrobial activity in the biofilm formation.

To analyze the effect of peptides **1026**, **RJK2**, and **R-FV7-I16** in biofilm detachment of Xff IVIA 5387.2, a quantification of cells by qPCR including the biofilm attached, biofilm detached, and planktonic cells was carried out. One hundred microliters of the peptides **1026**, **RJK2**, or **R-FV7-I16** were mixed with 900 μl of a X. fastidiosa suspension to a final peptide concentration of 50 μM in each well of a 24-well microplate. Non-treated wells were included as controls by substituting the volume of peptide with sterile water. A total of three replicates were made for treatment in each experiment. Two independent experiments were performed. Microplates were incubated at 28°C for 5 days under continuous shaking (120 rpm). Planktonic cells were recovered into tubes and centrifuged at 13,000 rpm for 10 min. Biofilm detached cells were recovered from the rinsing water by transferring the content of each well into a tube and centrifuging the mixture at 13,000 rpm for 10 min. This operation was repeated a total of six times and all the washes were collected in the same tube. Biofilm attached cells were recovered from each well by adding 1 ml of PBS, scrapping the attached cells with an inoculation loop, transferring them into a tube, and centrifuging them at 13,000 rpm for 10 min. All the pellets were suspended with PBS to a total volume of 1 ml. DNA extraction was performed for each sample and DNA samples were analyzed in duplicate by a TaqMan-based qPCR as previously described in the bactericidal activity experiments of this study.

Dose–effect relationship of **BP525**, **1037**, and **R-FV7-I16** on biofilm inhibition was determined. They were tested at 0, 6.3, 12.5, 25, and 50 μM against Xff IVIA 5387.2 as described in this study. Three replicates of 10 wells were made for each peptide and concentration. For dose–response modeling in inhibition of biofilm formation, percentage of biofilm inhibition (B_i_) was calculated according to the formula: B_i_ = 1 - (Oi/Oc) × 100, where Oi is the OD_595_ of the treatment and Oc is the OD_595_ of the non-treated control.

### Effect of Peptide Infiltration on Tobacco Leaves

Peptides were evaluated for their effect upon infiltration on tobacco leaves as previously described (Nadal et al., 2012). Briefly, tobacco plants (Nicotiana tabacum) were grown from seed in a heated glasshouse and used between 20 and 30 days old. Using a syringe, 100 μl of peptide solutions of 50, 100, and 150 μM were infiltrated into the mesophyll of fully expanded tobacco leaves (previously wounded with a needle). Six independent inoculations were carried out in a single leaf, and three independent inoculations were performed per peptide and concentration randomly distributed in different leaves and plants. Control infiltrations with water (negative control) or melittin (M2272, Merck, Madrid, Spain) (positive control) at the same molar concentrations were performed. Plants were kept at standard greenhouse conditions for 48 h. Peptide’s leaf infiltration effect was measured as the lesion diameter.

### Hemolytic Activity

The hemolytic activity of peptides was used as an indication of its toxicity, according to the current literature in this field (Montesinos et al., 2012; Inui Kishi et al., 2018). It was assessed by determining hemoglobin release from erythrocyte suspensions of horse blood (5% vol/vol) (SR0050C, Thermo Fisher Scientific, Spain) as previously described (Badosa et al., 2007). Briefly, peptides were solubilized in TRIS buffer and mixed with cleaned 10-fold diluted horse erythrocytes. The final peptide concentrations tested were 150, 250, and 375 μM. The percentage of hemolysis (H) was calculated using the equation: H = 100 × [(Op - Ob)/(Om - Ob)], where Op is the optical density at 540 nm for a given peptide concentration, Ob for the buffer, and Om for the melittin positive control.

### Data Analysis

Specific growth rates were estimated based on the slope of the growth curve (ln OD_600_ vs. time) at the exponential phase (Supplementary Figure 1). They were determined between 1 and 3 days for IVIA 5387.2 and Temecula strains, between 1 and 4 days for IVIA 5770 and CFBP 8173 strains, and between 3 and 6 days for DD1 and IVIA 5901.2 strains. The doubling time for each strain was calculated using the formula ln2/specific growth rate. To test the significance of the effect of strain on the parameters presented in Table 3, a one-way analysis of variance (ANOVA) was used. In all cases, means were separated according to the Duncan’s test at a p-value of < 0.05 (IBM SPSS Statistics for Windows, Version 25.0 released on 2017 by IBM Corp, Armonk, NY, United States).

**TABLE 3:** Growth, biofilm formation, and susceptibility of the Xylella fastidiosa strains to an antibacterial peptide (**BP171**) and an antibiofilm compound (NAC).

Subsp.^1^	Strain	Kinetic growth parameters^2^		Biofilm formation^3^	Bactericidal-BP171^4^		Antibiofilm-NAC^5^
					
		AUGC	Doubling time (h)	OD_595_ max	Reduction in viability (LogN_0_/N)		Biofilm formation (Ratio T/NTC)
					12.5 μM	3.1 μM	50 μM
Xff	Temecula	0.87 ± 0.02 d	20.26 ± 0.78 b	0.89 ± 0.07 a	3.18 ± 0.18 d	1.08 ± 0.12 c	0.94 ± 0.02 c
	IVIA 5387.2	0.78 ± 0.02 c	14.14 ± 0.81 a	1.59 ± 0.07 b	2.77 ± 0.02 c	0.87 ± 0.02 b	0.72 ± 0.01 b
	IVIA 5770	0.56 ± 0.02 b	26.27 ± 0.93 c	2.29 ± 0.10 c	2.52 ± 0.16 b	0.89 ± 0.08 b	0.97 ± 0.01 cd
Xfp	DD1	0.23 ± 0.01 a	124.51 ± 27.67 *	2.39 ± 0.14 c	0.91 ± 0.07 a	0.12 ± 0.08 a	0.42 ± 0.05 a
Xfm	CFBP 8173	1.20 ± 0.02 e	19.41 ± 1.57 b	0.93 ± 0.10 a	3.77 ± 0.16 e	1.53 ± 0.04 d	0.99 ± 0.02 cd
	IVIA 5901.2	0.19 ± 0.03 a	30.30 ± 3.46 d	0.80 ± 0.07 a	2.45 ± 0.04 b	1.89 ± 0.03 e	1.00 ± 0.04 d

*^1^Xff, X. fastidiosa subsp. fastidiosa; Xfp, X. fastidiosa subsp. pauca; Xfm, X. fastidiosa subsp. multiplex.*

^2^Kinetic growth parameters (area under the growth curve [AUGC] and doubling time). Values are the means of three replicates of 10 wells plus the confidence interval (α = 0.05). Means of kinetic growth parameters sharing the same letters are not significantly different (p < 0.05) according to the Duncan’s test. *DD1 was excluded of the statistical analysis of the doubling time due to extremely different behavior compared to the other strains.

*^3^Biofilm formation after 7 days (OD_595_ max). Values are the means of three replicates of 10 wells plus the confidence interval (α = 0.05). Means sharing the same letters are not significantly different (p < 0.05) according to the Duncan’s test.*

*^4^The reduction in viability was calculated as Log N_0_/N where N_0_ is 10^7^ CFU/ml of a non-treated control and N is CFU/ml of the treatment. Values are the means of three replicates plus the confidence interval (α = 0.05). Means sharing the same letters are not significantly different (p < 0.05) according to the Duncan’s test.*

*^5^All values are represented as a ratio between the OD_595_ obtained after the treatment (T) and the OD_595_ of a non-treated control (NTC). Values are the means of three replicates of 10 wells plus the confidence interval (α = 0.05). Means sharing the same letters are not significantly different (p < 0.05) according to the Duncan’s test.*

Also, to test the significance of the effect of peptides, peptide concentration, and time in the experiments, one-way ANOVA was performed. In all cases, means were separated according to the Duncan’s test (p < 0.05).

Data on peptide dose-biofilm inhibition were adjusted to a Michaelis-Menten model to determine the maximum biofilm inhibition (B_i_max) and the median effective dose (ED_50_):


$$Y=a\,\frac{X}{b+X}$$

where, *a* is the B_*i*_max and *b* is the ED_50_ (Waghu et al., 2018).

Principal components analysis (PCA) was used to evaluate singularities among the tested peptides to select the ones with the best biological profile (IBM SPSS Statistics for Windows, Version 25.0 released on 2017 by IBM Corp, Armonk, NY, United States). PCA was performed using 31 peptides on five variables: (i) bactericidal activity as the reduction in viability of *Xff* IVIA 5387.2, (ii) antibiofilm activity as the ratio of biofilm formation of *Xff* IVIA 5387.2, (iii) planktonic cells as the ratio of planktonic cells of *Xff* present after the peptide treatment, (iv) hemolytic activity as the percentage of hemoglobin release from erythrocyte suspensions of horse blood, and (v) leaf infiltration effect as the lesion diameter on tobacco leaves.

## Results

### Selection, Design, and Synthesis of the Peptides

This work was centered on identifying peptides active against *X. fastidiosa* (Table 1) and potential candidates included were: (i) sequences already reported with activity against *X. fastidiosa*, such as magainin 2 and indolicidin, and (ii) sequences with high activity against Gram-negative bacteria and/or with antibiofilm activity as well as with low toxicity, including **RR4-OH**, **LJK2**, **RIJK2**, **KR-12-a5**, **SB056**, **HP1404**, **HP1404 T1-D**, **HP1404 T1E**, **AamAP1**, **AamAP-S1**, **IDR-1018**, **HH15**, **1026**, **1029**, **1036**, **1037**, **FV7**, and **R-FV7-I16**. The structure of these peptides was used as template to design 11 new sequences. The new sequences were designed by reducing the peptide length [magainin 2(1–10)], replacing the Trp residues by D-Phe (**BP525**), incorporating an acyl group (**BP526** to **BP529**), preparing the amidated C-terminal analogs (**RR2-NH_2_**, **RR3-NH_2_**, and **RR4-NH_2_**), replacing D-amino acids by their L-enantiomers (**RJK2**), or replacing a Lys by an Arg (**AamAP-R**). These modifications have been reported to increase the antimicrobial activity of peptides (Guell et al., 2011; Cutrona et al., 2015; Vasilchenko et al., 2017; Oliveras et al., 2021).

These 31 peptides were manually synthesized following a standard Fmoc/^*t*^Bu strategy. They were obtained in excellent HPLC purities (93->99%), except for magainin 2 (64%), and their identity was confirmed by mass spectrometry (Supplementary Table 1).

### Growth and Biofilm Formation of *Xylella fastidiosa* Strains

Six *X. fastidiosa* strains belonging to three subspecies were characterized in relation to their capacity for growth and biofilm formation (Table 3 and Supplementary Figure 1). *Xfm* CFBP 8173 reached a higher area under the growth curve (AUGC, 1.2) than the other strains. The AUGC of *Xff* strains IVIA 5770, IVIA 5387.2, and Temecula was 0.56, 0.78, and 0.87, respectively, showing a similar growth curve. On the contrary, the two strains of *Xfm* had a different growth pattern and both *Xfm* IVIA 5901.2 and *Xfp* DD1 showed a poor growth, reaching AUGC values of 0.19 and 0.23, respectively. Regarding doubling time, the *Xfp* DD1 strain was the one that showed the most extreme behavior. Specifically, *Xfp* DD1’s doubling time ranged between 8.81 and 4.11 times larger compared to the other studied strains.

Biofilm formation was measured as OD_595_ after being dyed with crystal violet. *Xff* Temecula, *Xfm* CFBP 8173, and *Xfm* IVIA 5901.2 generated the lowest amount of biofilm, with OD_595_ values ranging from 0.8 to 0.93. *Xff* IVIA 5387.2 formed an intermediate amount of biofilm (OD_595_ of 1.59), whereas *Xfp* DD1 and *Xff* IVIA 5770 formed the highest amount of biofilm (OD_595_ ranging from 2.29 to 2.39).

**FIGURE 1:**
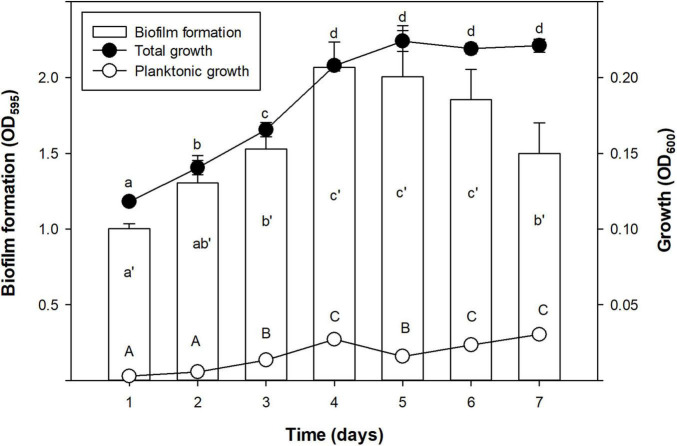
Time course of biofilm formation (OD_595_), total growth (OD_600_), and planktonic growth (OD_600_) of *X. fastidiosa* subsp. *fastidiosa* IVIA 5387.2. Values are the means of three replicates of 10 wells, and error bars represent the confidence interval (α = 0.05). Hyphenated letters correspond to the means comparison of biofilm formation, lowercase letters to total growth, and capital letters to planktonic growth. Means sharing the same letters within the same parameter are not significantly different (*p* < 0.05) across time according to the Duncan’s test.

Growth and biofilm formation kinetics of *Xff* IVIA 5387.2 was assessed (Figure 1). Total growth (including biofilm and planktonic cells) was characterized by a first stage of linear increase until the fourth day, followed by a stationary phase. Biofilm formation increased until a maximum at the fourth day and then it started to decrease on the seventh. Planktonic cells grew monotonically for the whole experiment. Interestingly, the highest values of planktonic cells were achieved with the decrease in biofilm during the stationary phase of growth.

### Bactericidal Susceptibility to Peptides and Effect of *N*-acetyl-L-cysteine on Biofilm Formation in *Xylella fastidiosa* Strains

Suitability of the v-qPCR method to quantify viable cells of different *X. fastidiosa* strains was analyzed. Standard curves obtained for the strains showed efficiencies ranging from 81 to 98.4% and the method had enough sensitivity to detect a minimum of 10^3^ CFU/ml of viable cells when mixed with dead cells (Supplementary Table 2 and Supplementary Figure 2).

Then, the susceptibility of six *X. fastidiosa* strains to the peptide **BP171** was assessed at 3.1 and 12.5 μM (Table 3) using the v-qPCR method. The bactericidal activity of the peptide was clearly dependent on the strains. Globally, *Xfm* CFBP 8173 displayed the highest reduction in viability followed closely by *Xfm* IVIA 5901.2 and *Xfp* DD1 was the most resistant strain to the peptide at both concentrations. IVIA 5387.2, IVIA 5770, and Temecula showed an intermediate resistance.

The effect of NAC on biofilm formation of *X. fastidiosa* strains was also assessed (Table 3). The effect was measured as a ratio between the OD_595_ values of treated and non-treated wells. NAC treatment affected the biofilm formation of *Xff* IVIA 5387.2 and *Xfp* DD1 with a ratio of 0.72 and 0.42, respectively. In contrast, it did not affect significantly the strains *Xff* Temecula, *Xfm* CFBP 8173, *Xff* IVIA 5770, and *Xfm* IVIA 5901.2 that showed ratios ranging from 0.94 and 1. Considering all of the above, *Xff* IVIA 5387.2 was selected in subsequent experiments as it showed intermediate susceptibility to the peptides.

### Bactericidal Activity

**FIGURE 2:**
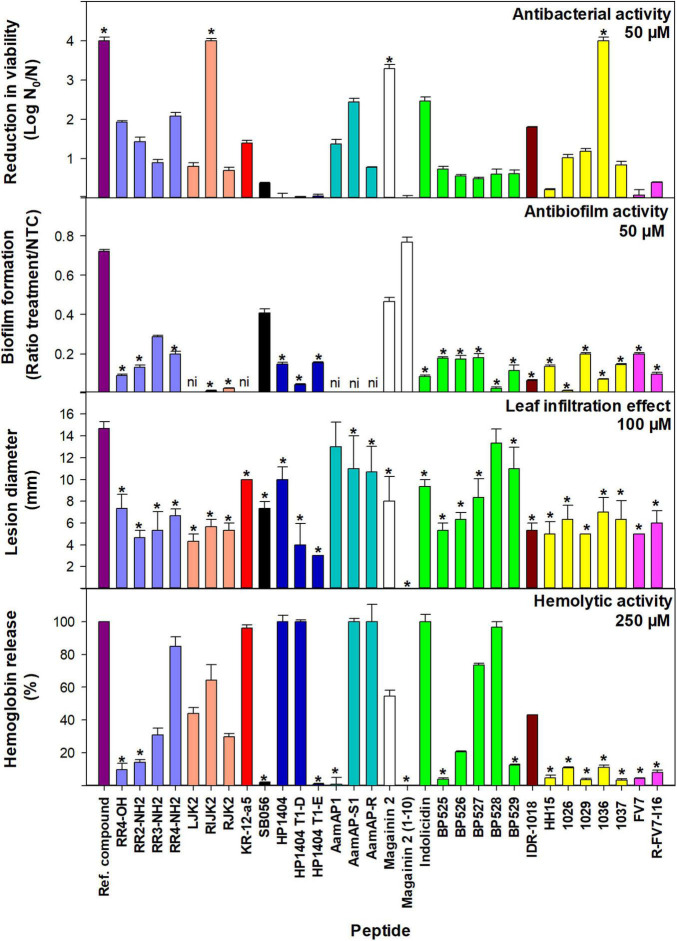
Bactericidal and antibiofilm activity against *X. fastidiosa* subsp. *fastidiosa* IVIA 5387.2, tobacco leaf infiltration effect, and hemolytic activity of peptides. Values are the means of three replicates and error bars represent the confidence interval (α = 0.05). Each color represents a different peptide family. The asterisk (*) indicates the peptides that have the best values for each activity according to the Duncan’s test (*p* < 0.05). The reference compounds used were cecropin B for bactericidal activity, NAC for antibiofilm activity, and melittin for tobacco leaf infiltration effect and hemolytic activity. For antibiofilm activity, peptides with very high bactericidal activity were diluted (magainin 2 at 12.5 μM and **RIJK2** and **1036** at 3.1 μM) and **LJK2**, **KR-12-a5**, **SB056**, **AamAP1**, **AamAP-S1**, and **AamAP-R** were not included (ni) since they affected *X. fastidiosa*’s growth.

The bactericidal activity of the 31 peptides was tested at 50 μM against *Xff* IVIA 5387.2 (Figure 2). Peptides were classified into five statistically different groups (Supplementary Table 3). **RIJK2**, **1036,** magainin 2, and the reference peptide cecropin B were highly active, leading to more than 3 log reduction of cell viability. **RR4-NH_2_**, **AamAP-S1**, and indolicidin exhibited high activity with 2 to 3 log reduction of cell viability. Seven peptides showed moderate activity with 1 to 2 log reduction of cell viability. Twelve peptides had low activity with a 0.3 and 1 log reduction and seven peptides showed very low activity with less than 0.3 log.

Peptides that showed very high bactericidal activity against *X. fastidiosa* were further tested at lower concentrations, 12.5 and 3.1 μM (Supplementary Table 3). At 12.5 μM, **1036** was the most active peptide with a higher log reduction than cecropin B (3.48 vs. 3.19). At this concentration, **RIJK2** and magainin 2 displayed similar activity with 2.34 and 2 log reduction, respectively. At 3.1 μM, except for magainin 2 that was poorly active, the other peptides exhibited log reductions ranging from 1.89 to 2.13. In contrast with the other peptides, **RIJK2** was similarly active at both concentrations.

### Antibiofilm Activity

**FIGURE 3:**
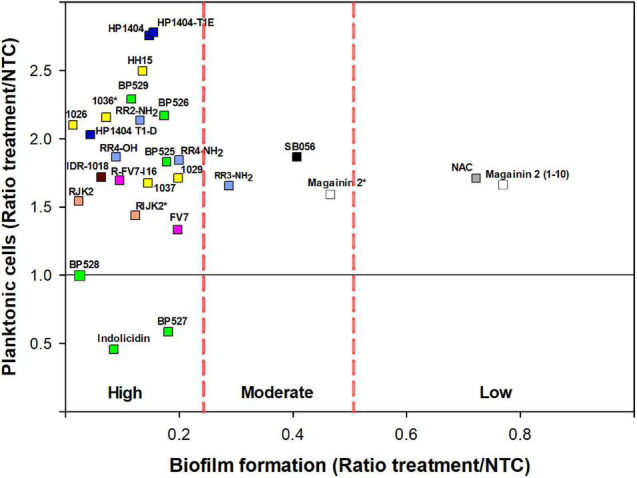
Effect of peptides on biofilm formation and planktonic cells on *X. fastidiosa* subsp. *fastidiosa* IVIA 5387.2. Peptide concentrations were 50 μM except for magainin 2 (12.5 μM), and **1036** and **RIJK2** (3.1 μM) which are marked with an asterisk (*). NAC was used as reference. Each color within the symbols represents a different peptide family. Values are the means of three replicates of 10 wells. Peptides were classified by antibiofilm activity using the Duncan’s test (*p* < 0.05) and were labeled as high, moderate, and low antibiofilm activity.

The antibiofilm activity of 31 peptides was determined against *Xff* IVIA 5387.2 at 50 μM (Figures 2, 3 and Supplementary Table 3). **RIJK2** and **1036** were tested at 3.1 μM and magainin 2 was tested at 12.5 mM to minimize the influence of their antimicrobial activity in the biofilm formation. Peptides that affected *X. fastidiosa*’s growth were not included in Figures 2, 3, specifically **LJK2**, **KR-12-a5**, **SB056**, **AamAP1**, **AamAP-S1**, and **AamAP-R**. Peptides were classified into three major groups according to their antibiofilm activity. Twenty out of 25 peptides exhibited high antibiofilm activity (ratio of biofilm formation from 0.01 to 0.2), peptide **1026** being the most active. It is worth mentioning that peptides **1036** and **RIJK2** showed a high antibiofilm activity despite being tested at 3.1 μM (ratio of biofilm formation of 0.07 and 0.12, respectively). Three peptides had moderate activity (ratio from 0.29 to 0.47) and two displayed low activity (ratio from 0.72 to 0.77) (Supplementary Table 3).

**FIGURE 4:**
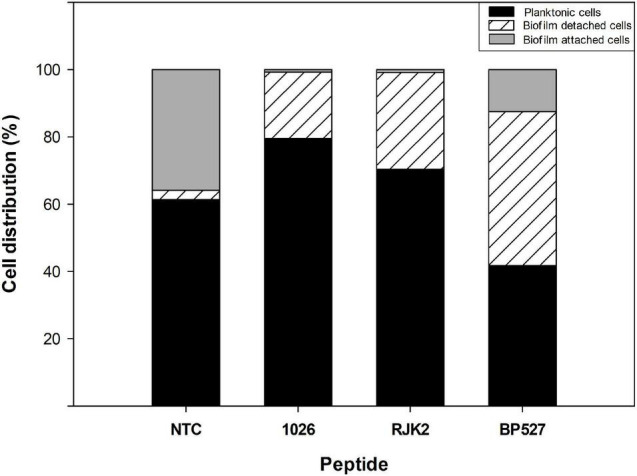
Effect of peptides **1026**, **RJK2**, and **BP527** on the percentage of planktonic cells and biofilm detached and attached cells of *X. fastidiosa* subsp. *fastidiosa* IVIA 5387.2 compared with a non-treated control (NTC). The experiment was performed at 50 μM. The total cells quantified in all treatments ranged between 1.63 × 10^8^ and 1.68 × 10^8^ CFU/ml.

Nevertheless, peptides with high antibiofilm activity exhibited a high variability in the ratio of planktonic cells that ranged from 0.46 to 2.78 (Figure 3). Peptides **1026**, **RJK2**, and **BP527**, which showed a high antibiofilm activity but exhibited a different ratio of planktonic cells, were further tested at 50 μM for their effect on biofilm formation of *Xff* IVIA 5387.2 (Figure 4). Planktonic, biofilm attached, and biofilm detached cells were quantified with qPCR. Biofilm detached cells correspond to a transition between biofilm attached cells and planktonic cells. A total amount of 1.6 × 10^8^ to 1.7 × 10^8^ CFU/ml of *Xff* IVIA 5387.2 were quantified in the wells in all treatments after 5 days of incubation. In non-treated cells, 35.9% of biofilm attached cells was observed together with only 2.8% of biofilm detached cells and 61.3% of planktonic cells. The exposure of *X. fastidiosa* to peptides reduced the biofilm formation and influenced biofilm attachment. In the treatment with peptide **1026**, only 0.7% of attached biofilm cells was observed, 19.8% were biofilm detached cells, and 79.5% planktonic cells. **RJK2** had a similar activity to **1026**. In contrast, **BP527** had less antibiofilm activity with 12.5% of attached biofilm cells, 45.7% of detached biofilm cells, and only 41.7% of planktonic cells observed. Therefore, the peptides differentially affected the balance between the attachment and detachment of biofilm cells.

**FIGURE 5:**
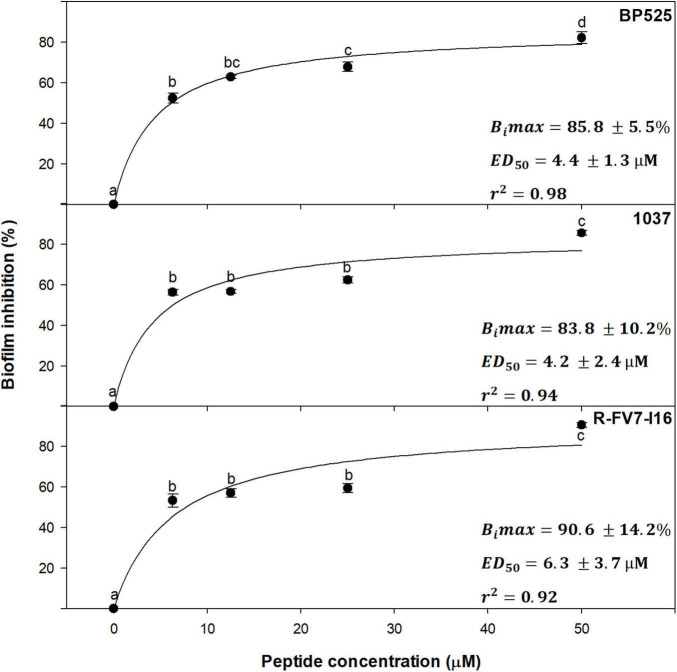
Dose–effect relationship of selected peptides on biofilm inhibition in *X. fastidiosa* subsp. *fastidiosa* IVIA 5387.2. Values are the means of three replicates of 10 wells, and error bars represent the confidence interval (α = 0.05). Means sharing the same letters are not significantly different (*p* < 0.05) according to the Duncan’s test. The line represents the Michaelis–Menten curve adjusted with the data points. B_*i*_max corresponds to maximum biofilm inhibition and ED_50_ corresponds to the median effective dose, which are indicated in each panel for each peptide. The coefficient of determination (*r*^2^) is also included in each panel.

Dose–effect relationship between peptide concentration and inhibition of biofilm was studied with a selection of peptides against *Xff* IVIA 5387.2. Peptides **BP525**, **1037**, and **R-FV7-I16** that belong to different families and showed high antibiofilm activity were selected (Figure 5). A direct relationship between peptide concentration and biofilm inhibition was observed, following a typical saturation kinetics that fitted well to a Michaelis-Menten model (*r*^2^ = 0.98, 0.94, and 0.92 for **BP525**, **1037**, and **R-FV7-I16**, respectively). All three peptides behaved similarly and their inhibitory activity increased rapidly between 0 and 3.1 μM, and remained stable from 12.5 to 50 μM. *B_*i*_max* of **R-FV7-I16, BP525**, and **1037** was estimated as 90.6% (±14.2), 85.8% (±5.5), and 83.3% (±10.2), respectively. *ED*_50_ was 4.2 ± 2.4, 4.4 ± 1.3, and 6.3 ± 3.7 μM for **1037**, **BP525**, and **R-FV7-I16**, respectively.

### Leaf Infiltration Effect on Tobacco Plants and Hemolytic Activity

The effect of the peptides on eukaryotic cells was assessed on tobacco leaves and erythrocytes. The peptide’s leaf infiltration effect was determined by infiltrating them into the mesophyll of tobacco plant leaves at 50, 100, and 150 μM (Supplementary Table 4). Melittin was used as a reference. Lesion diameter at 100 μM is shown in Figure 2. Melittin caused the highest lesion (14.7 mm), and except for **AamAP1** and **BP528**, peptides caused a lesion ranging from 0 to 11 mm, which was significantly lower than melittin.

Hemolytic activity of the peptides was determined on erythrocytes and compared to the reference peptide melittin (Supplementary Table 4). Percent hemolysis at 250 μM is shown in Figure 2. Fifteen out of the 31 peptides analyzed showed a hemolysis ≤14% and 5 exhibited a hemolysis between 20 and 44%.

### Grouping Peptides According to Their Biological Profile

**FIGURE 6:**
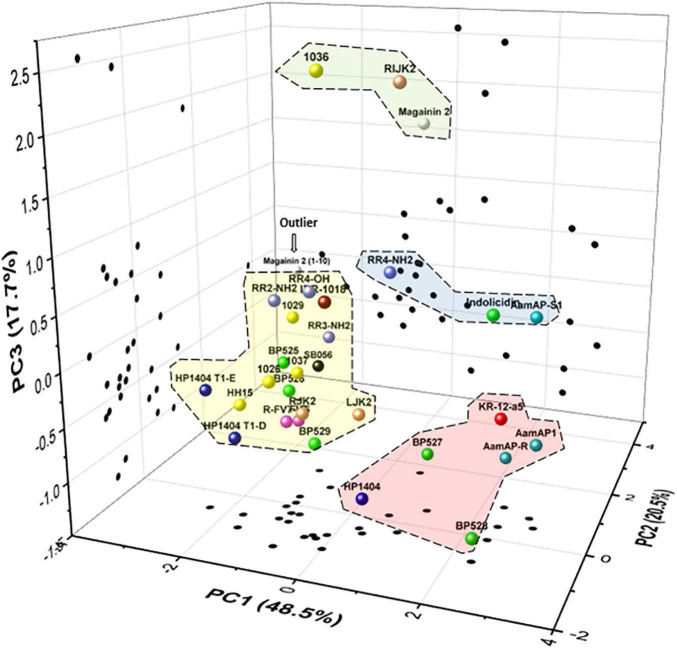
Principal components analysis (PCA) of the 31 peptides. Scatter plot of the peptides within the three axes including PC1 (leaf infiltration effect and hemolysis), PC2 (antibiofilm activity), and PC3 (bactericidal activity). Scatter plot projections of the peptides on the planes PC1 vs. PC2, PC2 vs. PC3, and PC1 vs. PC3 are also included (black dots). Discontinued lines correspond to groups of peptides according to their biological profile.

Five variables were selected for the biological profile analysis of the peptides (bactericidal activity, antibiofilm activity, planktonic cell presence, hemolytic activity, and leaf infiltration effect) to group peptides with a PCA. The first three principal components (PCs) accounted for 48.5, 20.5, and 17.7%, respectively, of the total variation in the dataset. Therefore, the three-dimensional scatter plot of the peptides is a good approximation as it represents 86.7% of the total variation of the data (Figure 6). The PC1 axis represents the variables leaf infiltration effect and hemolytic activity. The PC2 axis reflects the antibiofilm activity. The PC3 axis represents the bactericidal activity. Less toxic peptides have low values in PC1, peptides with higher antibiofilm activity have low values in PC2, and highly bactericidal peptides have high values in PC3.

In the PCA, four major groups and an outlier were identified. The first group was composed of **1036**, **RIJK2**, and magainin 2, which have high bactericidal activity, high antibiofilm activity, and moderate toxicity. The second group was formed by **RR4-NH_2_**, indolicidin, and **AamAP-S1,** which had moderate bactericidal activity, high antibiofilm activity, and moderate to high toxicity. The third group was represented by **KR-12-a5**, **AamAP1**, **AamAP-R**, **BP527**, and **HP1404** and had low bactericidal activity, high antibiofilm activity, and high toxicity. The fourth group was composed of all the other peptides except for magainin 2(1–10) and had low/moderate bactericidal activity, high/moderate antibiofilm activity, and low toxicity. Magainin 2(1–10) behaves differently from all the other peptides and it was considered as an outlier.

## Discussion

*X. fastidiosa* is a highly relevant plant-pathogenic bacterium in the European Union due to the high field productivity losses that it causes, which may dampen the local economy (Ferguson et al., 2017; EPPO, 2019; Schneider et al., 2020). Its main mechanism of pathogenicity is biofilm formation that may lead to the host death. Due to the impact of this pathogen, many strategies have been researched to control the diseases caused by *X. fastidiosa*. In general, promising results were obtained in reducing disease severity but no strategy was able to completely cure infected plants (Amanifar et al., 2016; Dongiovanni et al., 2017; Scortichini et al., 2018; Bruno et al., 2021). In this context, antimicrobial peptides, such as cecropin B, magainin 2, indolicidin, and **BP178**, have been previously reported to display antibacterial activity against *X. fastidiosa* (Li and Gray, 2003; Kuzina et al., 2006; Baró et al., 2020a, b). Although the number of reported antimicrobial peptides active against this bacterium are scarce, these examples pave the way to search for new candidates. Moreover, peptide sequences with antibiofilm activity against this pathogen have not been reported. Nevertheless, peptides able to affect biofilm formation have been described against other Gram-negative bacteria, and these sequences could be considered as potential candidates to be tested against *X. fastidiosa*. Thus, in this paper, the above sequences were taken as the basis for the design and identification of new peptides with bactericidal or antibiofilm activity against *X. fastidiosa*.

To assess the activity of peptides against *X. fastidiosa*, a strain displaying moderate values regarding growth, biofilm formation, and susceptibility to antimicrobial peptides was selected among six strains (*Xfp* DD1, *Xfm* CFBP 8173, *Xff* Temecula, *Xff* IVIA 5387.2, *Xff* IVIA 5770, and *Xfm* IVIA 5901.2). These strains belong to three of the major subspecies found in the Mediterranean area (*pauca*, *fastidiosa* and *multiplex*). These subspecies are more or less specific to a particular host range and climate conditions, so it would be expected that they displayed different behaviors in growth, biofilm formation, and susceptibility to antimicrobial compounds as it has been previously reported (Baldi and La Porta, 2017; Denancé et al., 2017, 2019).

In the present study, the strains differed greatly in all the evaluated parameters accordingly to other studies, which also observed noticeable differences between other *X. fastidiosa* strains regarding growth and biofilm formation (Feil and Purcell, 2001; Guilhabert and Kirkpatrick, 2005) and susceptibility to antimicrobial peptides (Baró et al., 2020a, b). Specifically, *Xff* Temecula, *Xff* IVIA 5387.2, and *Xff* IVIA 5770 displayed moderate values in growth, biofilm formation, and susceptibility to antimicrobial peptides. Interestingly, concerning these three strains, NAC, which was previously reported as an antibiofilm compound (Muranaka et al., 2013), only affected the biofilm formation of *Xff* IVIA 5387.2. In the case of *Xff* Temecula, the values of the growth parameters were similar to those previously reported in the literature (Guilhabert and Kirkpatrick, 2005; Sicard et al., 2020). Some of the other strains displayed a more extreme behavior. For example, *Xfm* CFBP 8173 exhibited a high growth and susceptibility to antimicrobial peptides, but low biofilm formation, which was not affected by NAC. In contrast, *Xfp* DD1 displayed a slow growth and formed abundant biofilm as observed in other studies (D’Attoma et al., 2020), but this biofilm was susceptible to NAC. Nevertheless, this strain is highly resistant to the tested antimicrobial peptides as it was previously reported (Baró et al., 2020b). *Xfm* IVIA 5901.2 exhibited a comparable growth pattern to that of *Xfp* DD1, but its susceptibility to antimicrobial peptides was similar to that of the other IVIA strains. Taking into account all these results, *Xff* IVIA 5387.2 was selected for next bactericidal and antibiofilm studies, because it presents a moderate behavior. Biofilm formation kinetics was assessed for this strain and maximum biofilm was formed between the 4th and 6th day in PD3 medium. This pattern was similar to the one previously reported for other *X. fastidiosa* strains (Cogan et al., 2013; Janissen et al., 2015). It corresponds to a typical biofilm formation kinetics, involving attachment of cells to a surface, EPS matrix secretion, biofilm formation, and biofilm maturation. Eventually, biofilm cells revert to a planktonic state and they are able to disperse.

The peptides tested in this study, including the reference peptide cecropin B, showed different degrees of bactericidal activity against *X. fastidiosa*, being classified into five major groups. The most interesting sequences were cecropin B, magainin 2, **1036**, and **RIJK2**, which displayed similar activity with a reduction in viability higher than 3.2 log. Cecropin B and magainin 2 had been previously reported as active against *X. fastidiosa* (Li and Gray, 2003; Kuzina et al., 2006). However, this is the first report on the activity of **1036** and **RIJK2** against *X. fastidiosa.* In fact, **1036** was previously reported to be active against *P. aeruginosa* and *B. cenocepacia*, and for **RIJK2,** only antibiofilm activity was described (De La Fuente-Núñez et al., 2012, 2015). It is interesting to highlight the difference in activity of **RIJK2** compared to their analogs. For example, **RIJK2** exhibited higher bactericidal activity than its all L-isomer **RJK2**, which could be ascribed to an increase in the stability of **RIJK2** due to the presence of D-amino acids into its sequence as previously described for other peptides (Guell et al., 2011; Molhoek et al., 2011; Carmona et al., 2013). Moreover, this increased stability of **RIJK2** could result in a reduction of its degradation susceptibility to the enzymes that *X. fastidiosa* secretes through outer membrane vesicles or through the type II secretion system (Ionescu et al., 2014; Rapicavoli et al., 2018; Feitosa-Junior et al., 2019). Nevertheless, more studies should be performed to confirm these observations. Most of the peptides tested in this work showed high antibiofilm activity against *X. fastidiosa.* Although some of them, such as **RR4-OH**, **RIJK2**, and **1036**, had been previously described to display antibiofilm activity against Gram-negative bacteria, this is the first time that their activity against *X. fastidiosa* is reported (De La Fuente-Núñez et al., 2012, 2015; Mohamed et al., 2017). Remarkably, we also identified peptides that had never been described to display antibiofilm activity. Among them, we found magainin 2 and indolicidin, only previously reported for their antibacterial activity against *X. fastidiosa* (Kuzina et al., 2006), and the newly designed peptides such as **BP526** and **RR4-NH_2_**.

Similarly to other antibiofilm peptides against human pathogens (Mishra and Guangshun, 2017; Park et al., 2019; Qi et al., 2020), the antibiofilm activity of **BP525**, **1037**, and **R-FV7-I16** showed a dose–effect relationship that fitted well with a Michaelis–Menten saturation curve. Interestingly, they showed low ED_50_ values, which means that low peptide concentrations already display high antibiofilm activity. This result suggests that, in a hypothetical plant application, the dilution of the peptides along the xylem vessels would not significantly affect their antibiofilm activity. Taking into account that these three peptides belong to different families of compounds, a similar behavior could be expected for the other peptides.

Interestingly, peptides that displayed high antibiofilm activity showed different patterns concerning the amount of planktonic cells detected during the screening of antibiofilm activity. This could indicate that the effect of these peptides on the biofilm formation may differ between them. The effect of **BP527**, **1026**, and **RJK2** in the biofilm formation was studied in detail. Peptides **1026** and **RJK2** exhibited antibiofilm activity, because most of *X. fastidiosa* cells remained in a planktonic stage preventing biofilm formation. In the case of **BP527**, antibiofilm activity was also observed, but less planktonic cells were detected. This could suggest that this peptide displayed its activity once the biofilm was formed by causing a detachment of biofilm cells. Therefore, this could indicate that peptides are able to affect biofilm formation of *X. fastidiosa* at different stages whether by directly preventing biofilm formation or by affecting the reversible/irreversible attachment phase. This behavior has been reported for other pathogens such as *P. aeruginosa* and *A. baumannii* when treated with **FLIP7** or ciprofloxacin (Macia et al., 2014; Gordya et al., 2017; Raheem and Straus, 2019). Nevertheless, further studies are needed to elucidate the exact role of these peptides in the inhibition of biofilm formation.

Regarding the hemolytic activity and the leaf infiltration effect of the peptides, it was not possible to establish a general pattern. In general, the peptides showed low hemolytic activity, and their effect upon infiltration on tobacco leaves was moderate and significantly lower than that of the reference peptide. It has to be taken into account that the effect observed in tobacco leaves might not necessarily be due to phytotoxicity, but it might be related to a hypersensitivity reaction caused by the peptides (Badosa et al., 2013). The least toxic families were those derived from **1036** and **FV7**. In the case of lipopeptides **BP526**-**BP528**, it is interesting to note that an increase of the fatty acid chain length led to an increase of the hemolysis. This correlation has been attributed to an increase of the peptide hydrophobicity that favors its affinity for the erythrocytes membrane (Malina and Shai, 2005; Oliveras et al., 2018).

To summarize, peptides with bactericidal and antibiofilm activity against *X. fastidiosa* and moderate toxicity have been identified. A PCA allowed to classify these peptides into four groups according to their distinct biological activity profile. An interesting group was composed by **1036**, **RIJK2**, and magainin 2 as they displayed dual activity (high bactericidal and antibiofilm activities) and moderate toxicity. Another group with many peptides displayed high antibiofilm activity, but low/moderate bactericidal activity and a low toxicity profile. Peptides **1036** and **RIJK2**, with dual activity against *X. fastidiosa* and moderate toxicity, would be the most promising ones as they may be able to simultaneously inhibit biofilm formation and kill *X. fastidiosa* cells. Nevertheless, peptides with only antibiofilm activity should also be taken into account as they may be able to eliminate the symptoms caused by the occlusion of the xylem vessels by *X. fastidiosa*. However, this could cause an increase of planktonic cells available for vector transmission (Ionescu et al., 2014). Moreover, these peptides could be used in combination with other antimicrobial peptides in order to reduce the planktonic cells. Therefore, in future experiments, the most promising peptides identified in the present work will be tested *in planta* in different hosts to determine their capability to control the diseases caused by *X. fastidiosa.*

## Data Availability Statement

The original contributions presented in the study are included in the article/Supplementary Material, further inquiries can be directed to the corresponding authors.

## Author Contributions

AB, EB, EM, MP, and LF obtained financial support. LM, LF, MP, EM, EB, and AB designed the research and analyzed the data. LM conducted and performed the experiments. All authors wrote, read, reviewed, and approved the final manuscript.

## Conflict of Interest

The authors declare that the research was conducted in the absence of any commercial or financial relationships that could be construed as a potential conflict of interest.

## Publisher’s Note

All claims expressed in this article are solely those of the authors and do not necessarily represent those of their affiliated organizations, or those of the publisher, the editors and the reviewers. Any product that may be evaluated in this article, or claim that may be made by its manufacturer, is not guaranteed or endorsed by the publisher.

## Supplementary Material

Supplementary Figure 1 Growth kinetics of the *X. fastidiosa* strains for 7 days. Values are the means of three replicates of ten wells, and error bars represent the confidence interval (α = 0.05).

Supplementary Figure 2 Standard curves were obtained from dilutions of a cellular suspension from *X. fastidiosa* subsp. *fastidiosa* IVIA 5387.2. The experiments were carried out with viable cells, dead cells, and a mixture of live cells with 10^6^ CFU/mL of dead cells. Cells were treated with PMAxx (black symbols) or not (white symbols) before DNA extraction. Three independent experiments were carried out and are represented as a circle, triangle and a square. The stripped background represents the detection limit of viable cells at C_*T*_ > 34.5.
