# Supplementary figures and images for: Antimicrobial Peptides With Antibiofilm Activity Against Xylella fastidiosa
Source: Front Microbiol. 2021 Nov 8;12:753874. doi: 10.3389/fmicb.2021.753874 (PMC8606745; doi:10.3389/fmicb.2021.753874)

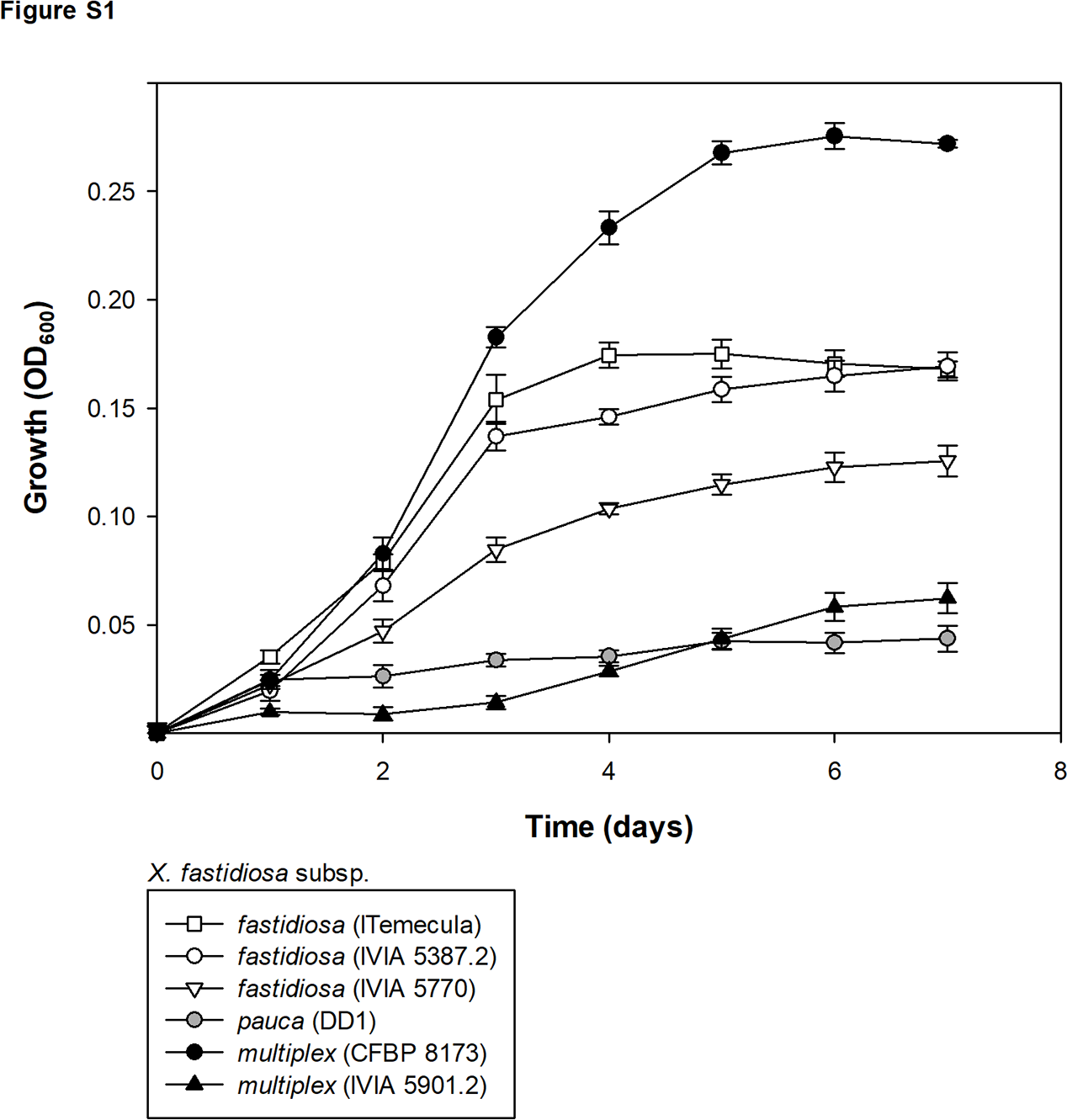

Supplement: Supplementary Figure 1 — Growth kinetics of the X. fastidiosa strains for 7 days. Values are the means of three replicates of ten wells, and error bars represent the confidence interval (α = 0.05). [file Image_1.TIF]

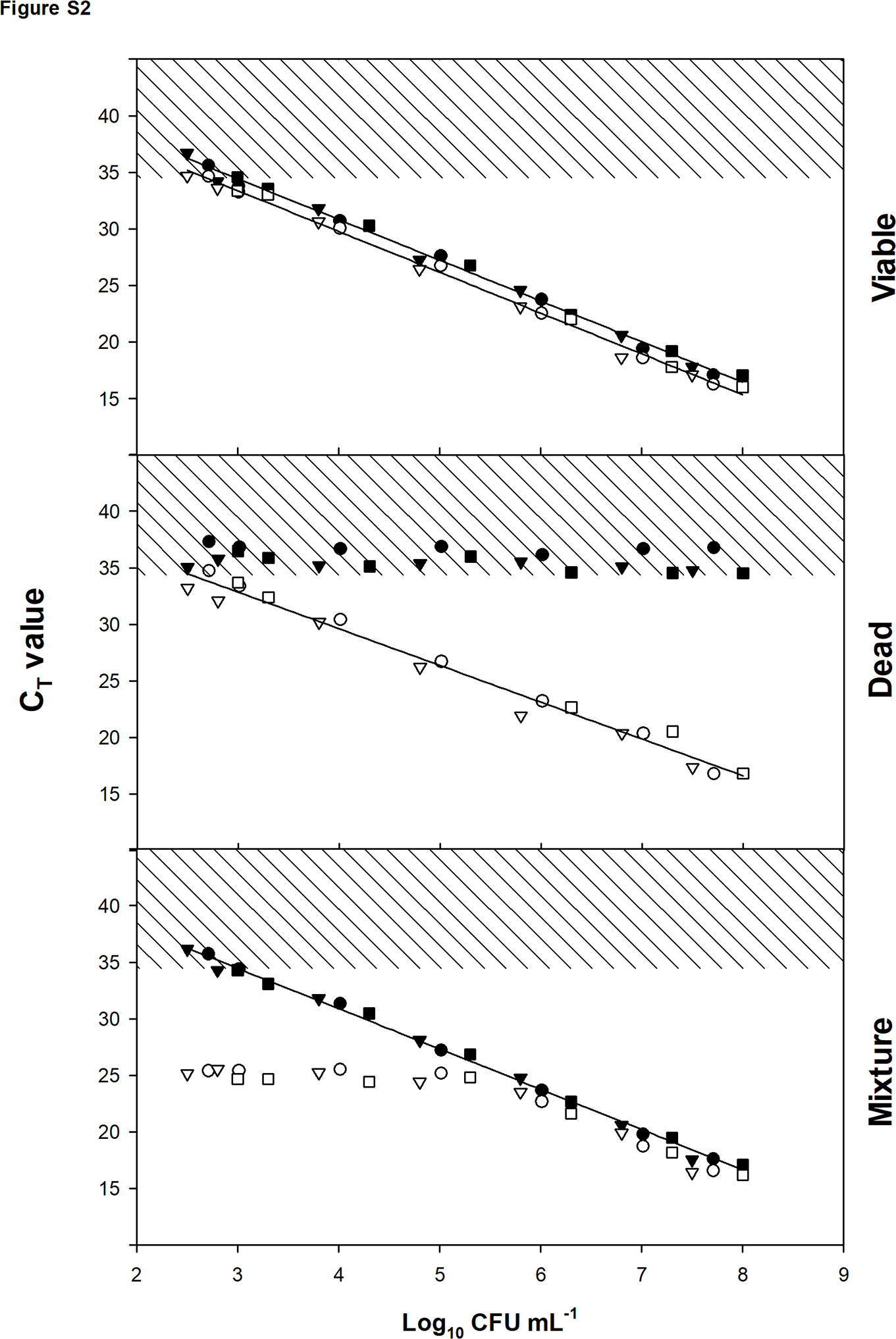

Supplement: Supplementary Figure 2 — Standard curves were obtained from dilutions of a cellular suspension from X. fastidiosa subsp. fastidiosa IVIA 5387.2. The experiments were carried out with viable cells, dead cells, and a mixture of live cells with 106 CFU/mL of dead cells. Cells were treated with PMAxx (black symbols) or not (white symbols) before DNA extraction. Three independent experiments were carried out and are represented as a circle, triangle and a square. The stripped background represents the detection limit of viable cells at CT > 34.5. [file Image_2.TIF]
