# Supplementary material for: Antimicrobial Peptides With Antibiofilm Activity Against Xylella fastidiosa
Source: Front Microbiol. 2021 Nov 8;12:753874. doi: 10.3389/fmicb.2021.753874 (PMC8606745; doi:10.3389/fmicb.2021.753874)
Supplement: Supplementary file 3 [file Table_1.DOCX]

Supplementary Material

**Table S1.** Sequences, retention time, purity and mass spectrometry data of the peptides

| **Code** | **Sequence^1^** | ***t*_R_ (min)^2^** | **Purity (%)^3^** | **HRMS (ESI)** | | |
| --- | --- | --- | --- | --- | --- | --- |
|  |  |  |  |  | **Calculated** | **Found** |
| **RR4-OH** | WLRRIKAWLRRIKA-OH | 5.51 | >99 | C_88_H_150_N_30_O_15_ [M+2H]^2+^ | 933.5943 | 933.5931 |
| **RR2-NH_2_** | WIRRIKKWIRRVHK-NH_2_ | 4.60 | >99 | C_93_H_158_N_34_O_14_ [M+2H]^2+^ | 987.6343 | 987.6316 |
| **RR3-NH_2_** | WLRRIKAWLRRKRK-NH_2_ | 4.93 | >99 | C_91_H_159_N_35_O_14_ [M+2H]^2+^ | 983.1397 | 983.1443 |
| **RR4-NH_2_** | WLRRIKAWLRRIKA-NH_2_ | 5.62 | >99 | C_88_H_151_N_31_O_14_ [M+2H]^2+^ | 933.1023 | 933.0994 |
|  |  |  |  |  |  |  |
| **LJK2** | VFWRRIRVWVIR-NH_2_ | 5.52 | >99 | C_82_H_130_N_27_O_12_ [M+2H]^2+^ | 843.0230 | 843.0209 |
| **RIJK2** | RIVWVRIRRWFV-NH_2_ | 5.71 | >99 | C_82_H_131_N_27_O_12_ [M+2H]^2+^ | 843.0230 | 843.0226 |
| **RJK2** | RIVWVRIRRWFV-NH_2_ | 5.93 | >99 | C_82_H_131_N_27_O_12_ [M+2H]^2+^ | 843.0230 | 843.0206 |
|  |  |  |  |  |  |  |
| **KR-12-a5** | KRIVKLILKWLR-NH_2_ | 5.97 | 97 | C_76_H_139_N_23_O_12_ [M+2H]^2+^ | 783.5497 | 783.5473 |
|  |  |  |  |  |  |  |
| **SB056** | WKKIRVRLSA-NH_2_ | 4.39 | >99 | C_58_H_104_N_20_O_11_ [M+2H]^2+^ | 628.9088 | 628.9107 |
|  |  |  |  |  |  |  |
| **HP1404** | GILGKLWEGVKSIF-NH_2_ | 7.02 | >99 | C_75_H_122_N_18_O_17_ [M+2H]^2+^ | 773.4612 | 773.4599 |
| **HP1404 T1-D** | ILKKLLKKVKSI-NH_2_ | 4.52 | >99 | C_68_H_134_N_18_O_13_ [M+2H]^2+^ | 705.5183 | 705.5164 |
| **HP1404 T1-E** | ILKKLLKKVKKI-NH_2_ | 4.56 | >99 | C_71_H_141_N_19_O_12_ [M+2H]^2+^ | 726.0498 | 726.0495 |
|  |  |  |  |  |  |  |
| **AamAP1** | FLFSLIPHAIGGLISAFK-NH_2_ | 7.39 | >99 | C_96_H_150_N_22_O_20_ [M+2H]^2+^ | 965.5693 | 965.5661 |
| **AamAP-S1** | FLFSLIPKAIGGLISAFK-NH_2_ | 7.77 | >99 | C_96_H_155_N_21_O_20_ [M+2H]^2+^ | 961.0873 | 961.0837 |
| **AamAP-R** | FLFSLIPRAIGGLISAFK-NH_2_ | 7.68 | >99 | C_96_H_155_N_23_O_20_ [M+2H]^2+^ | 975.0904 | 975.0867 |
|  |  |  |  |  |  |  |
| **Magainin 2** | GIGKFLHSAKKFGKAFVGEIMNS-NH_2_ | 5.96 | 64 | C_114_H_183_N_31_O_28_S [M+2H]^2+^ | 1233.6754 | 1233.6794 |
| **Magainin 2(1-10)** | GIGKFLHSAK-NH_2_ | 4.57 | >99 | C_49_H_83_N_15_O_11_ [M+2H]^2+^ | 528.8193 | 528.8183 |
|  |  |  |  |  |  |  |
| **Indolicidin** | ILPWKWPWWPWRR-NH_2_ | 6.59 | >99 | C_101_H_134_N_24_O_14_ [M+2H]^2+^ | 953.5250 | 953.5250 |
| **BP525** | ILPFKFPFFPFRR-NH_2_ | 6.25 | >99 | C_90_H_129_N_21_O_13_ [M+2H]^2+^ | 856.0034 | 856.0061 |
| **BP526** | C_3_H_7_CO-ILPFKFPFFPFRR-NH_2_ | 7.25 | >99 | C_94_H_135_N_21_O_14_ [M+2H]^2+^ | 891.0243 | 891.0210 |
| **BP527** | C_5_H_11_CO-ILPFKFPFFPFRR-NH_2_ | 7.62 | >99 | C_96_H_139_N_21_O_14_ [M+2H]^2+^ | 905.0400 | 905.0405 |
| **BP528** | C_11_H_23_CO-ILPFKFPFFPFRR-NH_2_ | 9.19 | >99 | C_102_H_151_N_21_O_14_ [M+2H]^2+^ | 947.0869 | 947.0851 |
| **BP529** | HOC_11_H_22_CO-ILPFKFPFFPFRR-NH_2_ | 7.76 | >99 | C_102_H_151_N_21_O_15_ [M+2H]^2+^ | 955.0844 | 955.0803 |
|  |  |  |  |  |  |  |
| **IDR-1018** | VRLIVAVRIWRR-NH_2_ | 5.27 | 93 | C_71_H_128_N_26_O_12_ [M+2H]^2+^ | 768.5097 | 768.5073 |
|  |  |  |  |  |  |  |
| **HH15** | KRFRIRVRVIRK-NH_2_ | 4.32 | 97 | C_73_H_138_N_30_O_12_ [M+2H]^2+^ | 813.5550 | 813.5521 |
| **1026** | VQWRIRVRVIKK-NH_2_ | 4.65 | 98 | C_73_H_132_N_26_O_13_ [M+2H]^2+^ | 790.5228 | 790.5200 |
| **1029** | KQFRIRVRV-NH_2_ | 4.29 | >99 | C_54_H_99_N_21_O_10_ [M+2H]^2+^ | 600.8936 | 600.8908 |
| **1036** | VQFRIRVRIVIRK-NH_2_ | 5.05 | 94 | C_77_H_142_N_28_O_14_ [M+2H]^2+^ | 841.5625 | 841.5613 |
| **1037** | KRFRIRVRV-NH_2_ | 4.28 | >99 | C_55_H_103_N_23_O_9_ [M+2H]^2+^ | 614.9149 | 614.9127 |
|  |  |  |  |  |  |  |
| **FV7** | FRIRVRV-NH_2_ | 4.53 | 95 | C_43_H_79_N_17_O_7_ [M+2H]^2+^ | 472.8169 | 472.8153 |
| **R-FV7-I16** | RFRRLFRIRVRVLKKI-NH_2_ | 4.93 | >99 | C_100_H_181_N_37_O_16_ [M+2H]^2+^ | 1078.2238 | 1078.2223 |

^1^ Underlined amino acids stand for the corresponding D-isomer

^2^ HPLC retention time

^3^ Percentage determined by HPLC at 220 nm
