# Supplementary material for: Antimicrobial Peptides With Antibiofilm Activity Against Xylella fastidiosa
Source: Front Microbiol. 2021 Nov 8;12:753874. doi: 10.3389/fmicb.2021.753874 (PMC8606745; doi:10.3389/fmicb.2021.753874)
Supplement: Supplementary file 4 [file Table_2.DOCX]

Supplementary Material

**Table S2.** Standard curves for the calibration of the qPCR assays obtained from dilutions of cellular suspensions from the *X. fastidiosa* strains.

| ***X. fastidiosa*** | **Strain** | **Condition^a^** | **PMA^a^** | **Standard Curve^a^** | **R^2^** | **Efficiency (%)** | **Sensitivity^b^** |
| --- | --- | --- | --- | --- | --- | --- | --- |
|  |  |  | | |  |  |  |
| subsp. *fastidiosa* | Temecula | Viable | + | C_T_ = -3.47 log CFU/mL + 44.03 | 0.99 | 94.1 | 36.99 |
|  |  |  | - | C_T_ = -3.50 log CFU/mL + 45.79 | 0.99 | 93.1 |  |
|  |  | Mixture | + | C_T_ = -3.64 log CFU/mL + 43.91 | 0.99 | 88.3 |  |
|  |  |  |  |  |  |  |  |
|  | IVIA 5387.2 | Viable | + | C_T_ = -3.60 log CFU/mL + 45.23 | 0.99 | 89.6 | 34.54 |
|  |  |  | - | C_T_ = -3.61 log CFU/mL + 44.21 | 0.99 | 89.3 |  |
|  |  | Mixture | + | C_T_ = -3.56 log CFU/mL + 45.14 | 0.99 | 90.9 |  |
|  |  |  |  |  |  |  |  |
|  | IVIA 5901.2 | Viable | + | C_T_ = -3.75 log CFU/mL + 45.91 | 0.99 | 84.8 | 35.24 |
|  |  |  | - | C_T_ = -3.74 log CFU/mL + 45.11 | 0.99 | 85.1 |  |
|  |  | Mixture | + | C_T_ = -3.45 log CFU/mL + 44.60 | 0.99 | 94.9 |  |
|  |  |  |  |  |  |  |  |
|  |  |  |  |  |  |  |  |
| subsp. *pauca* | DD1 | Viable | + | C_T_ = -3.29 log CFU/mL + 42.09 | 0.98 | 98.4 | 33.02 |
|  |  |  | - | C_T_ = -3.30 log CFU/mL + 40.77 | 0.99 | 98.0 |  |
|  |  | Mixture | + | C_T_ = -3.17 log CFU/mL + 41.19 | 0.99 | 92.8 |  |
|  |  |  |  |  |  |  |  |
|  |  |  |  |  |  |  |  |
| subsp. *multiplex* | IVIA 5770 | Viable | + | C_T_ = -3.66 log CFU/mL + 47.07 | 0.99 | 87.6 | 36.95 |
|  |  |  | - | C_T_ = -3.88 log CFU/mL + 47.10 | 0.99 | 81.0 |  |
|  |  | Mixture | + | C_T_ = -3.81 log CFU/mL + 47.77 | 0.99 | 83.0 |  |
|  |  |  |  |  |  |  |  |
|  | CFBP 8173 | Viable | + | C_T_ = -3.63 log CFU/mL + 44.66 | 0.99 | 88.6 | 35.31 |
|  |  |  | - | C_T_ = -3.73 log CFU/mL + 44.86 | 0.99 | 85.4 |  |
|  |  | Mixture | + | C_T_ = -3.74 log CFU/mL + 45.34 | 0.99 | 85.0 |  |

^a^ The standard curves were performed with viable cells at different CFU/mL or a mixture of live cells at different CFU/mL with 10^6^ UFC/mL of dead cells. Three independent experiments were carried out for each linear regression. Cells were treated with PMA (+) or not (-) before DNA extraction.

^b^ C_T_ value at which viable and dead cells cannot be differentiated.
