# Supplementary material for: Antimicrobial Peptides With Antibiofilm Activity Against Xylella fastidiosa
Source: Front Microbiol. 2021 Nov 8;12:753874. doi: 10.3389/fmicb.2021.753874 (PMC8606745; doi:10.3389/fmicb.2021.753874)
Supplement: Supplementary file 5 [file Table_3.docx]

Supplementary Material

**Table S3.** Bactericidal and antibiofilm activity of the compounds against *X. fastidiosa* subsp. *fastidiosa* IVIA 5387.2.

| **Code** | **Bactericidal activity^1^** | | | | | | | | |  | **Antibiofilm activity** | | | | | | |
| --- | --- | --- | --- | --- | --- | --- | --- | --- | --- | --- | --- | --- | --- | --- | --- | --- | --- |
|  | **50 µM** | | |  | **12.5 µM** | |  | **3.1 µM** | |  | **Planktonic cells^2^** | |  | **Biofilm formation^3^** | | | |
|  | **Reduction in viability**  **(Log N_0_/N)** | | **Level** |  | **Reduction in viability**  **(Log N_0_/N)** | |  | **Reduction in viability**  **(Log N_0_/N)** | |  |  |  |  |  | | |  |
|  |  |  |  |  |  |  |  |  |  |  | **Ratio (treatment/NTC)** | |  | **Ratio (treatment/NTC)** | | **Level** |  |
| **Cecropin B** | 4.00 ± 0.10 | s | Very high |  | 3.19 ± 0.06 | c |  | 2.13 ± 0.10 | c |  | nd | nd |  | nd | nd | nd |  |
| ***N*-Acetyl-L-cysteine** | 0.12 ± 0.01 | ab | Very low |  |  |  |  |  |  |  | 1.71 ± 0.03 | def |  | 0.72 ± 0.01 | o | Low |  |
| **RR4-OH** | 1.94 ± 0.03 | n | Moderate |  |  |  |  |  |  |  | 1.87 ± 0.10 | defg |  | 0.09 ± 0.01 | cdefgh | High |  |
| ***RR2-NH_2_*** | 1.43 ± 0.12 | m | Moderate |  |  |  |  |  |  |  | 2.13 ± 0.07 | fgh |  | 0.13 ± 0.01 | fghij | High |  |
| ***RR3-NH_2_*** | 0.89 ± 0.09 | j | Low |  |  |  |  |  |  |  | 1.66 ± 0.09 | def |  | 0.29 ± 0.01 | l | Moderate |  |
| ***RR4-NH_2_*** | 2.08 ± 0.09 | p | High |  |  |  |  |  |  |  | 1.84 ± 0.05 | defg |  | 0.20 ± 0.01 | k | High |  |
| **LJK2** | 0.80 ± 0.10 | hij | Low |  |  |  |  |  |  |  | - | - |  | - | - | - |  |
| **RIJK2** | 4.00 ± 0.06 | s | Very high |  | 2.35 ± 0.07 | b |  | 2.08 ± 0.03 | c |  | 1.43 ± 0.09* | cd |  | 0.12 ± 0.01* | efghi | High |  |
| ***RJK2*** | 0.70 ± 0.08 | gh | Low |  |  |  |  |  |  |  | 1.54 ± 0.07 | de |  | 0.02 ± 0.00 | ab | High |  |
| **KR-12-a5** | 1.40 ± 0.07 | m | Moderate |  |  |  |  |  |  |  | - | - |  | - | - | - |  |
| **SB056** | 0.38 ± 0.03 | c | Low |  |  |  |  |  |  |  | 1.87 ± 0.19 | defg |  | 0.41 ± 0.02 | m | Moderate |  |
| **HP1404** | 0.00 ± 0.12 | a | Very low |  |  |  |  |  |  |  | 2.75 ± 0.02 | i |  | 0.15 ± 0.01 | ghijk | High |  |
| **HP1404 T1-D** | 0.04 ± 0.00 | a | Very low |  |  |  |  |  |  |  | 2.03 ± 0.30 | efgh |  | 0.04 ± 0.00 | abcd | High |  |
| **HP1404 T1-E** | 0.05 ± 0.04 | a | Very low |  |  |  |  |  |  |  | 2.78 ± 0.10 | i |  | 0.15 ± 0.00 | hijk | High |  |
| **AamAP1** | 1.38 ± 0.12 | m | Moderate |  |  |  |  |  |  |  | - | - |  | - | - | - |  |
| **AamAP-S1** | 2.45 ± 0.09 | q | High |  |  |  |  |  |  |  | - | - |  | - | - | - |  |
| ***AamAP-R*** | 0.78 ± 0.01 | hij | Low |  |  |  |  |  |  |  | - | - |  | - | - | - |  |
| **Magainin 2** | 3.29 ± 0.11 | r | Very high |  | 2.07 ± 0.12 | a |  | 0.49 ± 0.10 | a |  | 1.59 ± 0.13* | def |  | 0.47 ± 0.02* | n | Moderate |  |
| ***Magainin 2(1-10)*** | 0.00 ± 0.06 | a | Very low |  |  |  |  |  |  |  | 1.67 ± 0.07 | def |  | 0.77 ± 0.02 | o | Low |  |
| **Indolicidin** | 2.46 ± 0.11 | q | High |  |  |  |  |  |  |  | 0.46 ± 0.07 | a |  | 0.09 ± 0.01 | bcdefg | High |  |
| ***BP525*** | 0.74 ± 0.06 | hi | Low |  |  |  |  |  |  |  | 1.83 ± 0.12 | defg |  | 0.18 ± 0.01 | ijk | High |  |
| ***BP526*** | 0.56 ± 0.03 | ef | Low |  |  |  |  |  |  |  | 2.17 ± 0.02 | fgh |  | 0.17 ± 0.02 | ijk | High |  |
| ***BP527*** | 0.49 ± 0.04 | de | Low |  |  |  |  |  |  |  | 0.58 ± 0.12 | ab |  | 0.18 ± 0.02 | ijk | High |  |
| ***BP528*** | 0.61 ± 0.12 | fg | Low |  |  |  |  |  |  |  | 0.99 ± 0.06 | bc |  | 0.03 ± 0.01 | abc | High |  |
| ***BP529*** | 0.62 ± 0.09 | fg | Low |  |  |  |  |  |  |  | 2.29 ± 0.03 | ghi |  | 0.12 ± 0.03 | efghi | High |  |
| **IDR-1018** | 1.80 ± 0.01 | o | Moderate |  |  |  |  |  |  |  | 1.72 ± 0.07 | def |  | 0.06 ± 0.01 | abcde | High |  |
| **HH15** | 0.21 ± 0.02 | b | Very low |  |  |  |  |  |  |  | 2.50 ± 0.05 | hi |  | 0.14 ± 0.01 | fghijk | High |  |
| **1026** | 1.03 ± 0.07 | k | Moderate |  |  |  |  |  |  |  | 2.10 ± 0.05 | efgh |  | 0.01 ± 0.00 | a | High |  |
| **1029** | 1.18 ± 0.08 | l | Moderate |  |  |  |  |  |  |  | 1.71 ± 0.04 | def |  | 0.20 ± 0.01 | k | High |  |
| **1036** | 4.00 ± 0.09 | s | Very high |  | 3.48 ± 0.13 | d |  | 1.89 ± 0.07 | b |  | 2.16 ± 0.06* | fgh |  | 0.07 ± 0.00* | abcdef | High |  |
| **1037** | 0.84 ± 0.09 | ij | Low |  |  |  |  |  |  |  | 1.67 ± 0.05 | def |  | 0.15 ± 0.00 | ghijk | High |  |
| **FV7** | 0.07 ± 0.14 | a | Very low |  |  |  |  |  |  |  | 1.33 ± 0.17 | cd |  | 0.20 ± 0.01 | jk | High |  |
| **R-FV7-I16** | 0.39 ± 0.01 | cd | Low |  |  |  |  |  |  |  | 1.70 ± 0.12 | def |  | 0.10 ± 0.01 | defgh | High |  |

^1^ Reduction in viability was calculated as Log N_0_/N where N_0_ is 10^7^ CFU/mL of a non-treated control and N is CFU/mL of the treatment. The confidence interval is shown (α=0.05). Compounds sharing the same letters are not significantly different according to the Duncan’s test (*p*<0.05).

^2^ Planktonic cells are represented as a ratio between the OD_600_ obtained after the treatment and the OD_600_ of a non-treated control. The confidence interval is shown (α=0.05). Compounds sharing the same letters are not significantly different according to the Duncan’s test (*p*<0.05).

^3^ Biofilm formation is represented as a ratio between the OD_595_ obtained after the treatment and the OD_595_ of a non-treated control. Peptides were tested at 50 µM except for magainin 2 (12.5 µM) and **RIJK2** and **1036** (3.1 µM) which are marked by an asterisk (*). The confidence interval is shown (α=0.05). Compounds sharing the same letters are not significantly different according to the Duncan’s test (*p*<0.05). nd, not determined; -, Peptides that affected *X. fastidiosa*’s growth were not included.
