# Supplementary material for: Antimicrobial Peptides With Antibiofilm Activity Against Xylella fastidiosa
Source: Front Microbiol. 2021 Nov 8;12:753874. doi: 10.3389/fmicb.2021.753874 (PMC8606745; doi:10.3389/fmicb.2021.753874)
Supplement: Supplementary file 6 [file Table_4.DOCX]

Supplementary Material

**Table S4.** Leaf infiltration effect and hemolysis of the compounds

|  | **Leaf infiltration effect (mm)^1^** | | | |  | **Hemolysis (%)^2^** | | | |
| --- | --- | --- | --- | --- | --- | --- | --- | --- | --- |
| **Code** | **50 µM** | **100 µM** | | **150 µM** |  | **150 µM** | **250 µM** | | **375 µM** |
| ***N*-Acetyl-L-cysteine** | 0 ± 0 | 0.00 ± 0.00 | a | 0.00 ± 0.00 |  | 0.5 ± 0.9 | 0.7 ± 1.1 | a | 1 ± 2 |
| **RR4-OH** | 3.67 ± 0.65 | 7.33 ± 1.31 | ghij | 16.67 ± 1.73 |  | 3.2 ± 1.0 | 9.8 ± 3.7 | def | 17.8 ± 0.3 |
| **RR2-NH_2_** | 3.33 ± 0.65 | 4.67 ± 0.65 | bcde | 9.33 ± 1.73 |  | 13.0 ± 1.7 | 14.0 ± 1.8 | g | 17.9 ± 0.4 |
| **RR3-NH_2_** | 3 ± 1.13 | 5.33 ± 1.73 | cdefg | 8.67 ± 1.31 |  | 19.0 ± 3.3 | 30.8 ± 4.2 | i | 37.3 ± 4.4 |
| **RR4-NH_2_** | 4.33 ± 0.65 | 6.67 ± 0.65 | efghi | 9.00 ± 1.13 |  | 64.3 ± 5.4 | 85.0 ± 5.7 | m | 100.0 ± 5.4 |
| **LJK2** | 3.33 ± 0.65 | 4.33 ± 0.65 | bcd | 5.00 ± 0.00 |  | 28.4 ± 1.9 | 43.9 ± 3.7 | j | 72.4 ± 8.9 |
| **RIJK2** | 2.33 ± 0.65 | 5.67 ± 0.65 | cdefg | 8.67 ± 1.31 |  | 35.1 ± 8.3 | 64.4 ± 9.4 | l | 71.0 ± 7.1 |
| **RJK2** | 5.67 ± 0.65 | 5.33 ± 0.65 | cdefg | 13.00 ± 0.00 |  | 19.2 ± 3.1 | 29.6 ± 2.2 | i | 50.2 ± 5.4 |
| **KR-12-a5** | 5.33 ± 0.65 | 10.00 ± 0.00 | kl | 9.67 ± 0.58 |  | 79.0 ± 0.6 | 96.1 ± 1.8 | o | 100.0 ± 1.7 |
| **SB056** | 4.33 ± 0.65 | 7.33 ± 0.65 | ghij | 8.33 ± 1.31 |  | 1.7 ± 0.2 | 1.8 ± 0.3 | a | 2.3 ± 0.7 |
| **HP1404** | 5.67 ± 0.65 | 10.00 ± 1.13 | kl | 16.67 ± 1.73 |  | 100.0 ± 0.5 | 100.0 ± 3.9 | o | 100.0 ± 0.7 |
| **HP1404 T1-D** | 3.33 ± 0.65 | 4.00 ± 1.96 | bc | 7.00 ± 3.20 |  | 0.3 ± 0.3 | 0.8 ± 1.1 | a | 1.3 ± 0.2 |
| **HP1404 T1-E** | 3.33 ± 0.65 | 3.00 ± 0.00 | b | 3.67 ± 1.31 |  | 0.8 ± 0.4 | 0.7 ± 0.4 | a | 1.5 ± 0.3 |
| **AamAP1** | 5.33 ± 1.31 | 13.00 ± 2.26 | m | 15.33 ± 1.73 |  | 100.00 ± 1.3 | 100.0 ± 4.1 | o | 100.0 ± 4.4 |
| **AamAP-S1** | 5.33 ± 0.65 | 11.00 ± 2.99 | l | 17.67 ± 0.65 |  | 100.00 ± 4.9 | 100.0 ± 2.1 | o | 100.0 ± 2.9 |
| **AamAP-R** | 5.33 ± 0.65 | 10.67 ± 2.36 | l | 13.67 ± 3.64 |  | 100.00 ± 4.3 | 100.0 ± 10.5 | o | 100.0 ± 3.1 |
| **Magainin 2** | 6.33 ± 0.58 | 8.00 ± 2.26 | hijk | 12.00 ± 2.99 |  | 35.2 ± 1.1 | 54.5 ± 3.7 | k | 71.1 ± 6.0 |
| **Magainin 2(1-10)** | 0 ± 0 | 0.00 ± 0.00 | a | 0.00 ± 0.00 |  | 0.0 ± 0.4 | 0.0 ± 0.2 | a | 0.0 ± 0.7 |
| **Indolicidin** | 4.33 ± 1.31 | 9.33 ± 0.65 | jkl | 10.00 ± 2.26 |  | 95.1 ± 5.0 | 100.0 ± 4.4 | o | 100.0 ± 2.5 |
| **BP525** | 4 ± 1.96 | 5.33 ± 0.65 | cdefg | 8.67 ± 0.65 |  | 3.1 ± 1.0 | 3.8 ± 0.8 | abc | 10.0 ± 5.9 |
| **BP526** | 3 ± 2.99 | 6.33 ± 0.65 | defghi | 8.00 ± 0.00 |  | 8.3 ± 0.7 | 20.4 ± 0.7 | h | 46.8 ± 3.3 |
| **BP527** | 5.67 ± 1.96 | 8.33 ± 1.73 | ijk | 11.33 ± 1.73 |  | 62.2 ± 4.4 | 73.6 ± 1.1 | l | 100.0 ± 2.6 |
| **BP528** | 6.67 ± 1.96 | 13.33 ± 1.31 | m | 14.00 ± 1.96 |  | 92.1 ± 5.7 | 96.6 ± 3.1 | n | 99.1 ± 8.0 |
| **BP529** | 6.33 ± 2.85 | 11.00 ± 1.96 | l | 12.00 ± 1.13 |  | 7.2 ± 0.3 | 12.5 ± 0.6 | fg | 39.0 ± 1.1 |
| **IDR-1018** | 3 ± 1.13 | 5.33 ± 0.65 | cdefg | 8.00 ± 1.00 |  | 35.6 ± 4.4 | 43.0 ± 0.2 | j | 45.9 ± 4.9 |
| **HH15** | 4.67 ± 0.65 | 5.00 ± 1.13 | bcdef | 6.00 ± 1.96 |  | 2.3 ± 0.3 | 4.5 ± 1.9 | abc | 4.0 ± 0.4 |
| **1026** | 4 ± 1.73 | 6.33 ± 1.31 |  | 7.67 ± 0.65 |  | 7.6 ± 0.8 | 10.8 ± 0.7 | efg | 14.5 ± 5.5 |
| **1029** | 0.33 ± 0.65 | 5.00 ± 0.00 | bcdef | 8.00 ± 1.13 |  | 2.3 ± 0.7 | 3.5 ± 0.7 | ab | 3.4 ± 0.6 |
| **1036** | 6 ± 1.13 | 7.00 ± 1.13 | fghi | 11.67 ± 0.65 |  | 6.5 ± 0.6 | 11.0 ± 1.6 | bcd | 17.3 ± 1.3 |
| **1037** | 4.33 ± 1.31 | 6.33 ± 1.73 | bcdef | 9.67 ± 1.31 |  | 2.4 ± 0.4 | 3.3 ± 0.7 | ab | 4.9 ± 0.2 |
| **FV7** | 4.67 ± 1.73 | 5.00 ± 0.00 | bcdef | 8.00 ± 2.99 |  | 3.7 ± 0.4 | 4.4 ± 0.3 | abc | 9.5 ± 1.4 |
| **R-FV7-I16** | 0.33 ± 0.65 | 6.00 ± 1.13 | cdefgh | 10.00 ± 2.99 |  | 6.3 ± 0.3 | 8.0 ± 1.3 | cde | 9.7 ± 0.6 |
| **Melittin** | 8.67 ± 1.73 | 14.67 ± 0.65 | m | 19.00 ± 1.13 |  |  | 100 | o |  |

^1^ Leaf infiltration effect determined as the lesion diameter (mm) in infiltrated tobacco leaves plus its confidence interval (α=0.05). Compounds sharing the same letters are not significantly different according to the Duncan’s test (*p*<0.05).

^2^ Percent hemolysis plus confidence interval (α=0.05). Compounds sharing the same letters are not significantly different according to the Duncan’s test (*p*<0.05).
